# Joint associations of folate, homocysteine and MTHFR, MTR and MTRR gene polymorphisms with dyslipidemia in a Chinese hypertensive population: a cross-sectional study

**DOI:** 10.1186/s12944-015-0099-x

**Published:** 2015-09-04

**Authors:** Wen-Xing Li, Wen-Wen Lv, Shao-Xing Dai, Ming-Luo Pan, Jing-Fei Huang

**Affiliations:** Institute of Health Sciences, Anhui University, Hefei, 230601 PR China; State Key Laboratory of Genetic Resources and Evolution, Kunming Institute of Zoology, Chinese Academy of Sciences, Kunming, 650223 PR China; School of Life Sciences, Anhui University, Hefei, 230601 PR China; KIZ-SU Joint Laboratory of Animal Models and Drug Development, College of Pharmaceutical Sciences, Soochow University, Suzhou, 215123 PR China; Collaborative Innovation Center for Natural Products and Biological Drugs of Yunnan, Kunming, 650223 PR China

**Keywords:** *MTHFR* C677T, *MTHFR* A1298C, *MTR* A2756G, *MTRR* A66G, Folate, Homocysteine, Serum lipids

## Abstract

**Background:**

Dyslipidemia is a well-established risk factor for cardiovascular disease. Serum lipids were affected by several gene polymorphisms, folate, homocysteine and other metabolite levels. We aim to investigate the effects of homocysteine metabolism enzyme polymorphisms (*MTHTR* C677T, *MTHFR* A1298C, *MTR* A2756G and *MTRR* A66G) and their interactions with folate, homocysteine on serum lipid levels in Chinese patients with hypertension.

**Methods:**

Participants were 480 hypertensive adults that enrolled in September to December 2005 from six different Chinese hospitals (Harbin, Shanghai, Shenyang, Beijing, Xi’an, and Nanjing). Known *MTHFR* C677T, *MTHFR* A1298C, *MTR* A2756G and *MTRR* A66G genotypes were determined by PCR-RFLP methods. Serum folate was measured by chemiluminescent immunoassay, homocysteine was measured by high-performance liquid chromatography, serum lipids parameters were determined by an automatic biochemistry analyzer, low-density lipoprotein was calculated by Friedewald’s equation. Unitary linear regression model was used to assess the associations of gene polymorphisms, folate and homocysteine on serum lipid profiles. Unconditional logistic regression model was applied to test the interactions of folate, homocysteine and gene polymorphisms on dyslipidemia.

**Results:**

No correlations between gene polymorphisms and homocysteine on serum lipid profiles. Compared with normal folate patients, patients with low folate showed higher odds of hypertriglyceridemia (OR = 2.02, 95 % CI: 1.25-3.25, P = 0.004) and low levels of high-density lipoprotein cholesterol (OR = 1.88, 95 % CI: 1.07-3.28, P = 0.027). Each of four gene polymorphisms (*MTHTR* C677T, *MTHFR* A1298C, *MTR* A2756G and *MTRR* A66G) combined with low folate showed higher odds of hypertriglyceridemia (P for trend: 0.049, 0.004, 0.007 and 0.005, respectively). *MTHFR* C677T and A1298C with low folate showed higher odds of low levels of high-density lipoprotein cholesterol (P for trend: 0.008 and 0.031).

**Conclusions:**

Low folate status and homocysteine metabolism gene polymorphisms (*MTHTR* C677T, *MTHFR* A1298C, *MTR* A2756G and *MTRR* A66G) may have a synergistic effect increased the incidence of dyslipidemia in Chinese hypertensive population.

## Background

Cardiovascular diseases (CVDs) are still a major cause of morbidity and mortality worldwide, which is expected to remain the same during the foreseeable future [1]. Dyslipidemia is a major risk factor for stroke, coronary artery disease, atherosclerosis and other CVDs [2–4]. Sun et al. showed that 64.4 % of rural Chinese adults had at least one type of abnormal lipid concentration [5]. The prevalence of dyslipidemia in Chinese was higher than that in American [6], Canadian [7], and Iranian population [8]. In addition, abnormal folate or homocysteine (Hcy) level was also exacerbated the incidence of CVDs [9, 10]. Recently, a large sample of clinical trials showed folate supplements effectively lower Hcy level and reduced the risk of stroke [11]. However, the potential mechanisms of folate, Hcy and serum lipid levels remain to be explored.

Our previous study found Hcy metabolism gene polymorphisms (*MTHFR* and *MTRR*) are significantly associated with serum lipid levels [12]. These findings suggest that there is an inherent correlation among folate, Hcy and lipid profiles. Additionally, several studies showed that medical nutrition therapy with folate intake significantly reduced serum lipids levels [13, 14]. Thus, we speculate that Hcy-metabolism gene polymorphisms associated with folate and Hcy interactively affect the incidence of dyslipidemia.

Several key enzymes, including methylenetetrahydrofolate reductase (MTHFR) [15], methionine synthase (MTR) [16] and methionine synthase reductase (MTRR) [17] are important in folate and Hcy metabolism and also in methylation reactions. MTHFR converts 5, 10-methylenetetrahydrofolate into 5-methyltetrahydrofolate and this reaction provide a methyl for homocysteine into methionine in the catalyzed by MTR [18]. MTR requires vitamin B_12_ (cobalamin) as a coenzyme. Over time, the cobalamin(I) cofactor of MTR will be oxidized to cobalamin(II) which leads to inactivation of MTR. And MTRR restore oxidized cobalamin(II) to CH_3_-cobalamin(III) in order to maintain the activity of MTR [17]. *MTHFR* C677T (rs1801133) is a common mutation of MTHFR and its suggested being an unfavorable factor for cardiovascular diseases [19, 20]. Yilmaz et al. showed that the C allele has a protective effect on blood lipid concentration and the T allele has a harmful effect when they are screening *MTHFR* C677T polymorphism in renal transplant patients [21]. However, another mutation *MTHFR* A1298C (rs1801131) seemed have no influences on serum lipids levels [12]. In hyperlipidemia patients, *MTR* A2756G (rs1805087) mutation was associated with a higher level of total cholesterol and low-density lipoprotein cholesterol [22]. *MTRR* A66G (rs1801394) is a susceptible marker of congenital heart defects [23], but the effect of this mutation on lipid profiles still no conclusion.

We hypothesized that homocysteine metabolism gene polymorphisms in combination with a low folate level or a high Hcy level may affect serum lipid profiles. In this study, we aim to explore the independent associations of folate, Hcy and these four gene polymorphisms (*MTHFR* C677T, *MTHFR* A1298C, *MTR* A2756G and *MTRR* A66G) on serum lipid profiles, as well as to evaluate the joint effect of gene, Hcy and genotypes on dyslipidemia in Chinese hypertensive patients.

## Results

### Clinical characteristics and genotype distributions

A total of 480 patients were recruited for this study. Since 55 patients missing the data of genotypes or lipids parameters were excluded, a total of 425 subjects were included in our final analysis. The average age of our patients was 56.7 ± 9.9 years, including 185 (43.5 %) men and 240 (56.5 %) women (Table 1). Genotype frequencies in the total population were showed in Table 2. Four gene polymorphisms (*MTHFR* C677T, *MTHFR* A1298C, *MTR* A2756G and *MTRR* A66G) in this population had no deviation from the Hardy-Weinberg equilibrium (P values were 0.885, 0.384, 0.937 and 0.400, respectively).

**Table 1:** Clinical and epidemiologic characteristics of population

Characteristic	Value
Age, year	56.7 ± 9.9
Sex	
Male	185 (43.5)
Female	240 (56.5)
Height, cm	162.7 ± 8.1
Weight, kg	68.2 ± 11.0
BMI, kg/m^2^	25.7 ± 3.4
SBP, mm Hg	153.9 ± 11.1
DBP, mm Hg	92.9 ± 8.1
Folate, nmol/L	13.7 ± 5.8
Hcy, μmol/L	15.1 ± 11.2
TC, mmol/L	5.00 ± 1.11
TAG, mmol/L	1.74 ± 1.24
HDL-C, mmol/L	1.31 ± 0.36
LDL-C, mmol/L	2.92 ± 0.79
Clinical Centers	
Ha’rbin	52 (12.2)
Shanghai	52 (12.2)
Shenyang	78 (18.4)
Beijing	95 (22.4)
Xi'an	71 (16.7)
Nanjing	77 (18.1)

*BMI* body mass index, *SBP* systolic blood pressure, *DBP* diastolic blood pressure, *Hcy* homocysteine, *TC* total cholesterol, *TAG* triacylglycerols, *HDL-C* high-density lipoprotein cholesterol, *LDL-C* low-density lipoprotein cholesterol

**Table 2:** Hardy-Weinberg equilibrium test of four genotypes

Genotype	Observed frequency	Expected frequency	*χ* ^2^	P
*MTHFR* C677T				
CC	108 (25.4)	107.3 (25.2)	0.021	0.885
CT	211 (49.6)	212.5 (50.0)		
TT	106 (24.9)	105.3 (24.8)		
*MTHFR* A1298C				
AA	299 (70.4)	296.5 (69.8)	0.759	0.384
AC	112 (26.4)	116.9 (27.5)		
CC	14 (3.3)	11.5 (2.7)		
*MTR* A2756G				
AA	348 (81.9)	347.9 (81.9)	0.006	0.937
AG	73 (17.2)	73.3 (17.2)		
GG	4 (0.9)	3.9 (0.9)		
*MTRR* A66G				
AA	137 (32.2)	141.2 (33.2)	0.708	0.400
AG	216 (50.8)	207.5 (48.8)		
GG	72 (16.9)	76.2 (17.9)		

### Associations of folate, Hcy and lipid profiles

The associations of serum folate and lipid profiles were showed in Table 3. Although folate may have no influence on serum TC and LDL-C, there was a significant negative correlation between folate and TAG before adjusted covariates (β (SE): -0.11 (0.01), P = 0.029). However, when adjusted several confounding factors this correlation disappeared. Furthermore, we observed a positive correlation between folate and HDL-C (crude β (SE): 0.15 (0.00), P = 0.002; adjusted β (SE): 0.14 (0.00), P = 0.002). We further used folate as a dichotomous variable to assess the influences of low folate on dyslipidemia (Table 4). Consistent with table three, patients with low folate showed higher odds of hypertriglyceridemia (OR (95 % CI): 2.02 (1.25-3.25), P = 0.004) and low levels of high-density lipoprotein cholesterol (OR (95 % CI): 1.88 (1.07-3.28), P = 0.027).

**Table 3:** Associations of folate level and serum lipid profiles

Parameters	Crude		Adjusted^b^	
	β (SE)^a^	P	β (SE)^a^	P
TC	0.01 (0.01)	0.836	0.06 (0.01)	0.235
TAG	-0.11 (0.01)	0.029	-0.07 (0.01)	0.151
HDL-C	0.15 (0.00)	0.002	0.14 (0.00)	0.002
LDL-C	-0.03 (0.01)	0.596	-0.00 (0.01)	0.984

*TC* total cholesterol, *TAG* triacylglycerols, *HDL-C* high-density lipoprotein cholesterol, *LDL-C* low-density lipoprotein cholesterol

^a^β = regression coefficient

^b^Adjusted for sex, age, clinical centers, height and weight

**Table 4:** Effect of low folate on serum lipids status

Folate status^a^	TC		OR (95 % CI)	P^b^	TAG		OR (95 % CI)	P^b^
	>5.18	≤5.18			>1.70	≤1.70		
High FA, %	71.5	72.3	Ref.		63.9	76.2	Ref.	
Low FA, %	28.5	27.7	0.87 (0.54-1.40)	0.569	36.1	23.8	2.02 (1.25-3.25)	0.004
	HDL-C		OR (95 % CI)	P^b^	LDL-C		OR (95 % CI)	P^b^
	<1.04	≥1.04			>3.37	≤3.37		
High FA, %	61.7	74.9	Ref.		71.3	72.3	Ref.	
Low FA, %	38.3	25.1	1.88 (1.07-3.28)	0.027	28.7	27.7	0.91 (0.56-1.48)	0.698

*TC* total cholesterol, *TAG* triacylglycerols, *HDL-C* high-density lipoprotein cholesterol, *LDL-C* low-density lipoprotein cholesterol

^a^High FA: serum folate level ≥ 10 nmol/L, Low FA: serum folate level < 10 nmol/L

^b^Adjusted for sex, age, clinical centers, height and weight

We also analyzed the associations of Hcy and these lipid profiles. However, we had no significant findings (data not show). And high Hcy status had no effect on the incidence of dyslipidemia (data not show).

### Associations of genotypes and lipid profiles

The correlations of these four gene polymorphisms and serum lipids levels were showed in Table 5. There was a marginal significance that the *MTRR* 66GG carriers had a higher serum total cholesterol level compared with 66AA carriers (β (SE): 0.14 (0.09), P = 0.054). We further analyzed the influences of gene polymorphisms on dyslipidemia risk, and the results were not reached significance (data not show).

**Table 5:** Associations of genotypes and serum lipid profiles

Genotype	TC			TAG		
	Mean ± SD	β (SE)^a^	P^b^	Mean ± SD	β (SE)^a^	P^b^
*MTHFR* C677T						
CC	5.01 ± 1.42	Reference		1.75 ± 1.06	Reference	
CT	4.94 ± 0.98	-0.05 (0.13)	0.337	1.75 ± 1.19	-0.01 (0.13)	0.799
TT	5.12 ± 1.00	0.02 (0.08)	0.811	1.69 ± 1.49	-0.03 (0.08)	0.701
*MTHFR* A1298C						
AA	4.97 ± 0.98	Reference		1.69 ± 1.15	Reference	
AC	5.10 ± 1.45	0.05 (0.12)	0.263	1.89 ± 1.48	0.06 (0.13)	0.231
CC	4.94 ± 0.83	0.01 (0.12)	0.846	1.56 ± 0.94	-0.03 (0.15)	0.633
*MTR* A2756G						
AA	5.02 ± 1.11	Reference		1.76 ± 1.30	Reference	
AG	4.92 ± 1.09	-0.00 (0.14)	0.966	1.62 ± 0.93	-0.02 (0.15)	0.698
GG	5.18 ± 2.23	0.02 (0.28)	0.714	1.73 ± 0.40	-0.02 (0.31)	0.703
*MTRR* A66G						
AA	4.82 ± 0.99	Reference		1.71 ± 1.25	Reference	
AG	5.03 ± 0.95	0.09 (0.10)	0.091	1.77 ± 1.30	-0.01 (0.13)	0.853
GG	5.27 ± 1.64	0.14 (0.09)	0.054	1.70 ± 1.03	-0.02 (0.08)	0.757
Genotype	HDL-C			LDL-C		
	Mean ± SD	β (SE)^a^	P^b^	Mean ± SD	β (SE)^a^	P^b^
*MTHFR* C677T						
CC	1.33 ± 0.36	Reference		2.87 ± 0.83	Reference	
CT	1.28 ± 0.35	-0.08 (0.04)	0.111	2.91 ± 0.80	0.01 (0.10)	0.801
TT	1.34 ± 0.39	-0.01 (0.02)	0.904	2.97 ± 0.74	0.04 (0.05)	0.550
*MTHFR* A1298C						
AA	1.30 ± 0.36	Reference		2.93 ± 0.77	Reference	
AC	1.31 ± 0.36	0.02 (0.04)	0.689	2.89 ± 0.84	-0.02 (0.09)	0.707
CC	1.39 ± 0.48	0.08 (0.05)	0.129	2.92 ± 0.82	0.00 (0.11)	0.993
*MTR* A2756G						
AA	1.31 ± 0.37	Reference		2.91 ± 0.78	Reference	
AG	1.28 ± 0.33	-0.02 (0.04)	0.658	2.91 ± 0.84	0.01 (0.10)	0.784
GG	1.33 ± 0.42	0.04 (0.09)	0.456	3.39 ± 0.85	0.06 (0.20)	0.288
*MTRR* A66G						
AA	1.27 ± 0.37	Reference		2.85 ± 0.79	Reference	
AG	1.32 ± 0.35	0.06 (0.04)	0.223	2.94 ± 0.80	0.06 (0.09)	0.303
GG	1.36 ± 0.37	0.07 (0.03)	0.274	2.98 ± 0.77	0.06 (0.06)	0.369

*TC* total cholesterol, *TAG* triacylglycerols, *HDL-C* high-density lipoprotein cholesterol, *LDL-C* low-density lipoprotein cholesterol

^a^β = regression coefficient

^b^Adjusted for sex, age, clinical centers, height and weight

### Interactions of folate, Hcy and genotypes on dyslipidemia

Compared with normal folate and wild-type carriers, those with low folate and mutant genotypes showed higher odds of hypertriglyceridemia (Table 6). In trend test, all these gene polymorphisms (*MTHFR* C677T, *MTHFR* A1298C, *MTR* A2756G and *MTRR* A66G) with low folate increased the prevalence of hypertriglyceridemia (interaction of *MTHFR* C677T and folate: P = 0.049; interaction of *MTHFR* A1298C and folate: P = 0.004; interaction of *MTR* A2756G and folate: P = 0.007; interaction of *MTRR* A66G and folate: P = 0.005, respectively). Table 7 showed the interactions of genotypes and low folate on low levels of high-density lipoprotein cholesterol. Compared with normal folate and *MTHFR* 677CC carriers, patients with both low folate and 677CT + TT genotype showed higher odds of low levels of HDL-C (OR (95 % CI): 2.67 (1.08-6.60), P = 0.033). Furthermore, low folate patients with the mutant allele of *MTHFR* A1298C showed a marginal significance higher incidence of low levels of HDL-C (OR (95 % CI): 2.52 (0.97-6.57), P = 0.059) than high folate patients carried 1298AA genotype. These findings were also confirmed by the trend test (interaction of *MTHFR* C677T and folate: P = 0.008; interaction of *MTHFR* A1298C and folate: P = 0.031, respectively). However, for interactions of genotypes and low folate on hypercholesterolemia and high levels of LDL-C, we have no significant findings.

**Table 6:** Interactions of genotypes and low folate with hypertriglyceridemia

Genotype	Folate status^a^	High TAG^b^	Low TAG^b^	OR (95 % Cl)	P^c^	Trend test	P^c^
*MTHFR* C677T							
CC	High FA	30 (20.8)	54 (19.2)	Reference		0.10 (0.02)	0.049
CT + TT	High FA	62 (43.1)	160 (56.9)	0.66 (0.38-1.16)	0.153		
CC	Low FA	11 (7.6)	13 (4.6)	1.44 (0.43-4.81)	0.555		
CT + TT	Low FA	41 (28.5)	54 (19.2)	1.44 (0.70-2.94)	0.325		
*MTHFR* A1298C							
AA	High FA	60 (41.7)	151 (53.7)	Reference		0.14 (0.02)	0.004
AC + CC	High FA	32 (22.2)	63 (22.4)	1.27 (0.74-2.17)	0.388		
AA	Low FA	38 (26.4)	50 (17.8)	2.23 (1.27-3.91)	0.005		
AC + CC	Low FA	14 (9.7)	17 (6.0)	2.51 (1.07-5.89)	0.035		
*MTR* A2756G							
AA	High FA	76 (52.8)	180 (64.1)	Reference		0.13 (0.02)	0.007
AG + GG	High FA	16 (11.1)	34 (12.1)	1.09 (0.55-2.16)	0.802		
AA	Low FA	41 (28.5)	51 (18.1)	2.05 (1.20-3.52)	0.009		
AG + GG	Low FA	11 (7.6)	16 (5.7)	1.92 (0.82-4.53)	0.134		
*MTRR* A66G							
AA	High FA	24 (16.7)	78 (27.8)	Reference		0.14 (0.02)	0.005
AG + GG	High FA	68 (47.2)	136 (48.4)	1.47 (0.84-2.57)	0.173		
AA	Low FA	17 (11.8)	18 (6.4)	3.39 (1.31-8.79)	0.012		
AG + GG	Low FA	35 (24.3)	49 (17.4)	2.51 (1.21-5.19)	0.013		

*TAG* triacylglycerols

^a^High FA: serum folate level ≥ 10 nmol/L, Low FA: serum folate level < 10 nmol/L

^b^High TAG: serum TAG > 1.70 mmol/L; Low TAG: serum TAG ≤ 1.70 mmol/L

^c^Adjusted for sex, age, clinical centers, height and weight

**Table 7:** Interactions of genotypes and low folate with low levels of high-density lipoprotein cholesterol (HDL-C)

Genotype	Folate status^a^	Low HDL-C^b^	High HDL-C^b^	OR (95 % Cl)	P^c^	Trend test	P^c^
*MTHFR* C677T							
CC	High FA	13 (13.8)	71 (21.5)	Reference		0.12 (0.02)	0.008
CT + TT	High FA	45 (47.9)	177 (53.5)	1.82 (0.87-3.81)	0.114		
CC	Low FA	7 (7.4)	17 (5.1)	1.13 (0.23-5.65)	0.883		
CT + TT	Low FA	29 (30.9)	66 (19.9)	2.67 (1.08-6.60)	0.033		
*MTHFR* A1298C							
AA	High FA	40 (42.6)	171 (51.7)	Reference		0.10 (0.02)	0.031
AC + CC	High FA	18 (19.1)	77 (23.3)	0.94 (0.48-1.82)	0.842		
AA	Low FA	24 (25.5)	64 (19.3)	1.59 (0.82-3.08)	0.172		
AC + CC	Low FA	12 (12.8)	19 (5.7)	2.52 (0.97-6.57)	0.059		
*MTR* A2756G							
AA	High FA	51 (54.3)	205 (61.9)	Reference		0.08 (0.02)	0.099
AG + GG	High FA	7 (7.4)	43 (13.0)	0.53 (0.21-1.32)	0.173		
AA	Low FA	27 (28.7)	65 (19.6)	1.66 (0.88-3.13)	0.116		
AG + GG	Low FA	9 (9.6)	18 (5.4)	1.73 (0.66-4.52)	0.263		
*MTRR* A66G							
AA	High FA	17 (18.1)	85 (25.7)	Reference		0.07 (0.02)	0.142
AG + GG	High FA	41 (43.6)	163 (49.2)	1.28 (0.66-2.49)	0.468		
AA	Low FA	19 (20.2)	16 (4.8)	4.66 (1.67-13.06)	0.003		
AG + GG	Low FA	17 (18.1)	67 (20.2)	1.52 (0.59-3.92)	0.382		

^a^High FA: serum folate level ≥ 10 nmol/L, Low FA: serum folate level < 10 nmol/L

^b^Low HDL-C: serum HDL-C < 1.04 mmol/L; High HDL-C: serum HDL-C ≥ 1.04 mmol/L

^c^Adjusted for sex, age, clinical centers, height and weight

We also carried out the analysis of interactions of high Hcy and genotypes with dyslipidemia profiles. Regrettably, no dyslipidemia odds were observed to be associated with high Hcy and mutant genotypes (data not show).

## Discussion

In the present study, we investigated the association of folate, Hcy and several gene polymorphisms (*MTHFR* C677T, *MTHFR* A1298C, *MTR* A2756G and *MTRR* A66G) on serum lipid profiles and assessed the interactions of folate, Hcy and genotypes with dyslipidemia. The results showed patients with folate deficiency showed higher odds of hypertriglyceridemia and low levels of HDL-C. Furthermore, these mutant genotypes combined with low folate may synergistically affect the incidence of hypertriglyceridemia and low levels of HDL-C. However, we haven’t found homocysteine directly or indirectly affected serum lipid profiles.

The mutation of *MTHFR* C677T is located in the catalytic domain of the enzyme, cause an alanine to valine substitution at position 222 of the enzyme, and result in a thermolabile enzyme [15, 24]. A previous study reported that homozygous mutation of 677TT reduced by approximately 70 % of the mean enzyme activity and the heterozygous mutation of 677CT reduced by approximately 35 % of the mean MTHFR activity [15]. It is no doubt that *MTHFR* C677T polymorphism was related to elevate plasma Hcy concentration and lower folate level [25–27]. Whereas its effect on lipid profiles is no conclusion. Frelut et al. showed *MTHFR* C677T significantly increased LDL-C level, and this mutant may also associate with higher total cholesterol and triacylglycerols levels, and lower HDL-C level, although it did not reach significance [28]. In polycystic ovary syndrome women, patients with 677CT genotype have higher serum total cholesterol and triacylglycerols than 677CC carriers [29]. In northern Chinese subjects with hyperlipidemia, individual carried *MTHFR* 677CT + TT genotypes showed higher triacylglycerols and total homocysteine compared with wild-type [22]. However, Chen et al. found no significant association between *MTHFR* C677T and lipid profiles (TC, TAG, HDL-C and LDL-C) in total population, but in subgroup analysis, the mutant T allele carriers have higher total cholesterol and low-density lipoprotein cholesterol than 677CC group in women [30]. In another Chinese clinical research, both plasma Hcy level and *MTHFR* C677T mutant have no association with hyperlipidemia or serum lipid levels [31]. In our study, *MTHFR* C677T may not affect serum lipid levels, and high homocysteine concentration also has no effect on serum lipid levels or dyslipidemia profiles. Furthermore, interaction of *MTHFR* C677T and Hcy also showed no influence on dyslipidemia. However, there was a trend for the combination of *MTHFR* 677CT + TT and low folate significantly increased the incidence of hypertriglyceridemia and low levels of HDL-C. Mikael et al. found that MTHFR deficiency affects apolipoprotein levels and leads to lipid deposition, in mice with MTHFR deficiency showed higher plasma TAG level than controls [32]. Based on the negative correlation between *MTHFR* C677T and folate level [33], and the detrimental effect of folate deficiency on lipid metabolism [34], we speculate that *MTHFR* C677T polymorphism and folate deficiency interactively increased the prevalence of dyslipidemia.

The mutation of *MTHFR* A1298C resulted the substitution of adenine to cytosine at nucleotide 1298 position, cause a glutamic acid to alanine substitution at position 429 of the peptide, is located in the regulatory domain named NADPH and S-adenosyl methionine binding site [24, 35]. Experimental results show that the A1298C mutation wasn’t caused a thermolabile enzyme [35]. Publications about the influence of *MTHFR* A1298C mutant on blood lipid profiles were relatively small. In a previous study, both *MTHFR* C677T and *MTHFR* A1298C polymorphisms not affect total cholesterol level [36]. Additionally, Chang et al. found no association between *MTHFR* C677T and *MTHFR* A1298C on blood lipid profiles (TC, TAG, HDL and LDL) [37]. These results were consisted with our findings, *MTHFR* A1298C independent not associated with serum lipids level and also have no effect on dyslipidemia profiles. However, low folate patients carried *MTHFR* 1298 AC + CC showed a higher risk of hypertriglyceridemia and low levels of HDL-C compared with the high folate patients with 1298AA genotype, although the latter was marginal significance. An *in vitro* experiment showed folate has a direct protective effect on LDL oxidation and its dose-related [38]. However, an animal experiment showed folate deficiency did not influence triacylglycerol levels [32]. Therefore, the potential mechanisms of the joint effect between *MTHFR* A1298C and folate deficiency on dyslipidemia need further research.

In our study, *MTR* A2756G polymorphism independently not affects serum lipid profile or dyslipidemia. However, the mutant allele with low folate may elevate the incidence of hypertriglyceridemia. The A2756G mutation in MTR that located at position 919 of the protein, results in substitution of glycine for aspartic acid [39]. It’s located in a domain of the protein that interacts with S-adenosyl methionine (SAM) and auxiliary proteins that are required for the reductive methylation and reactivation of the vitamin B_12_ cofactor, which can be inactivated by oxidation during catalysis [17, 40]. Therefore, it is possible that the mutant might impair the binding of SAM and/or auxiliary proteins [40] and increased plasma homocysteine concentration [41]. In hyperlipidemia patients, *MTR* 2756AG + GG carriers have higher total cholesterol and low-density lipoprotein cholesterol than those 2756AA carriers [22]. Additionally, the prevalence of 2756AG + GG genotypes in the combined hyperlipidemia group (hypercholesterolemia and hypertriglyceridemia) was significantly higher than that in the control group [22]. Regardless of some research found no association between *MTR* A2756G and folate level [25, 27], a study showed serum folate level, *MTHFR* 677CC + CT and *MTR* 2756AA genotypes have a significant interaction on total homocysteine concentration in pregnant woman [25]. Furthermore, individuals with low intake of folate, vitamin B_6_ and vitamin B_12_, the *MTHFR* 677 T allele and *MTR* 2756G allele were associated with a higher risk of breast cancer [42]. These results indicate that the mutation of *MTR* A2756G with other unfavorable factors may affect serum lipid profiles.

Our study showed a borderline significant that *MTRR* 66GG elevated serum TC level. Difference with our results, a previous study suggested that individual with *MTRR* 66GG genotype have lower serum TC and LDL-C level than 66AA carriers in Chinese hypertensive patients [12]. The mutation of *MTRR* A66G is the substitution of A for G at nucleotide 66 with a substitution of an isoleucine by a methionine. It has been suggested that the mutation is located in the putative flavin mononucleotide-binding domain of the MTRR enzyme that interacts with MTR [17] and thus disrupt the binding of MTRR to the MTR-cobalamin-complex, thereby decreasing the rate of homocysteine remethylation [39]. Our results showed *MTRR* 66AA patients with low folate had a 3.4-fold risk of hypertriglyceridemia and a 4.7-fold of high levels of LDL-C. Numerous studies showed *MTRR* A66G was a risk factor for congenital heart defects [23, 43, 44]. But the effect of this mutation on blood lipid profiles was lack of research. So we believe that the joint associations of *MTRR* A66G and folate with dyslipidemia are worthy to be further study.

Folate plays an important role in one-carbon metabolism. It serves as a catalytic substrate for the transfer of one-carbon units. When fed folate deficiency or methyl donor deficiency diets, rats showed lower hepatic folate, phosphocholine (PC) storage and higher plasma homocysteine, glycine, serine and threonine concentrations [45]. Furthermore, compared with control animals, rats with folate or methyl donor deficiency showed several protein regulation disorder and abnormal gene expression in liver [45]. Additionally, folate deficiency will elevate liver triacylglycerols level in spontaneously hypertensive rats [46]. These studies indicate that folate deficiency will damage the hepatic lipid homeostasis. Although short-term folate deficiency has weak impact on lipid metabolism, a long-term folate deficiency showed a marked hepatic lipid accumulation in rats [45, 47]. In our study, folate was associated with higher serum TAG and lower serum HDL-C level, patients with low folate showed higher odds of hypertriglyceridemia and low levels of HDL-C. Similar to our findings, Kim et al. found serum folate levels were significantly and positively correlated with HDL cholesterol and negatively with TAG, although the latter showed borderline significance [48]. In homocysteine metabolism, folate as a methyl donor that provides methyl for the synthesis of S-adenosyl methionine (SAM), SAM is a key intermediate in PC synthesis [49]. Decreased SAM level will increase S-adenosyl homocysteine (SAH), a competitive inhibitor of many methyltransferase reactions. So an important function of folate is maintenance the cellular SAM and SAH concentrations [34]. Therefore, folate deficiency will reduce PC synthesis resulting in accumulation of hepatic TAG that synthesis from phosphatidylethanolamine N-methyltransferase (PEMT) derived PC [34]. Furthermore, folate deficiency also reduced PEMT activity and choline kinase expression, and induced the expression of genes involved in hepatic lipid synthesis [34]. In oral contraceptive-treated rats, those with low dietary folate showed lower plasma HDL level and higher LDL level than control groups [50]. Another study showed higher serum folate level was associated with a lower level of LDL-C, higher levels of HDL-C and a lower LDL-C-C/HDL-C-ratio [51]. Elevated level of LDL is related to pathogenesis of atherosclerotic vascular disease whereas HDL has antioxidant effect that beneficial for against atherosclerosis and anti-inflammatory [3]. High Hcy was also suggested inducing hepatic cholesterol biosynthesis and lipid accumulation [52]. The liver is the main sites for lipids and Hcy metabolism. Hypomethylation associated with hyperhomocysteinemia was related to lipid accumulation in tissues, elevated Hcy increased SAH level thus inhibited PEMT and lower the production of PC from phosphatidylethanolamine [49]. An animal experiment showed a significant positive correlation between plasma Hcy and triacylglycerols in female mice [32].

Additionally, studies showed that serum lipid profiles were also regulated by vitamin B_6_ and vitamin B_12_. Fat oxidation requires carnitine and the endogenous synthesis of carnitine mainly occurs in the liver and kidneys and requires lysine, methionine and vitamin B_6_ [53]. Furthermore, vitamin B_6_ plays an important role in the desaturation and elongation of fatty acids, the mobilization of unsaturated fatty acids from triglycerides to phospholipids, and methylation of phospholipids [54]. Low vitamin B_6_ level was reported to impair carnitine synthesis and hence alter the lipid profiles. However, vitamin B_6_ supplementation was found to reduce the different components of the lipid profile by ~10 %, and significantly lowering TC and HDL-C level [53]. Vitamin B_12_ is a crucial constituent of the one carbon cycle and plays an important role in the homocysteine metabolism. It’s also a cofactor for the mitochondrial enzyme methyl malonyl CoA mutase and regulates the rate of long chain fatty acyl-CoA transfer into the mitochondria. Vitamin B_12_ deficiency leads to the accumulation of fatty acids in the cytosol thereby influencing lipid metabolic pathways [55]. Adaikalakoteswari et al. reported that vitamin B_12_ deficiency increased serum triglycerides and HDL level in Europeans and Indians with type 2 diabetes [56]. An animal experiment also showed the adverse effect of vitamin B_12_ deficiency on plasma lipid profiles and liver fatty acid levels [57]. However, Wasilewska et al. found no association between B vitamins (folate, vitamin B_6_ or vitamin B_12_) and lipid profiles in any examined group [58].

Therefore, we speculate that B vitamins, some metabolites and genotypes may synergistically affect the hepatic lipid metabolism and serum lipid profiles. However, the molecular mechanism of these metabolites under conditions of different gene polymorphisms on lipid profiles is not fully understood, and need for more extensive research.

## Conclusion and limitations

Our study demonstrated that there was a joint effect of homocysteine-metabolism gene polymorphisms (*MTHFR* C677T, *MTHFR* A1298C, *MTR* A2756G and *MTRR* A66G) and low folate on dyslipidemia in Chinese hypertensive patients. However, the underlying mechanism is not clear. Therefore, we believe that future researches could be more effective to elucidate these findings in a wider population.

Our study had several limitations. Firstly, this was a cross-sectional study that we can’t make a prediction about the incidence of dyslipidemia in the future, and the prevalence-incidence bias may also exist. Furthermore, our study sample was relatively small, and it may lack of effect when performed subgroup analysis. Next, serum lipid profiles may modify by vitamin B_6_ and vitamin B_12_, and we didn’t determine these B vitamins. Additionally, the limitation of generalization in our study was the study population only contained hypertensive adults, so these results need to be verified in a healthy population. In addition, serum lipid profiles may relate to dietary habits that we have not included.

## Methods

### Participants and procedures

This study was conducted using data collected in a previous study [59]. This was a multicenter, randomized, double-blind controlled trial in hypertensive Chinese adults. Details regarding “Study subjects”, “Randomization and double blinding”, “Data collection procedures”, and “Laboratory tests” have been previously described [59]. Totally, 480 patients with mild or moderate hypertension were recruited from six hospitals in different Chinese regions (Ha’erbin, Shanghai, Shenyang, Beijing, Xi’an, and Nanjing) from September to December 2005. All six hospitals were certified as clinical pharmacology centers by the State Food and Drug Administration in China. This study was approved by the Ethics Committee of Peking University First Hospital, Beijing, China. The purpose and procedures of the study were carefully explained to all participants, and written informed consent was obtained from each participant.

### Anthropometric measurements

The participants were invited to our clinic center at 8 am the next day after fasting overnight. After 60 min resting in a supine position, supine blood pressures were measured by using a mercury sphygmomanometer. No alcohol, cigarette smoking, coffee or tea was taken before the measurements. Blood pressure was measured by trained nurses. According to World Health Organization (WHO) standardized criteria, SBP was recorded at the appearance of sounds (first Korotkoff sounds) and DBP was recorded at the disappearance of sounds (fifth Korotkoff sounds). Three consecutive measurements were taken on the arms with 30s interval between replicates. If the difference between the measurements was more than 4 mm Hg, the patient was asked to rest for 5 min, and then repeated the measurements. In all of our analysis, the average of three consecutive blood pressure readings was used. Height was measured without shoes to the nearest 0.1 cm on a portable stadiometer. Weight was measured in light indoor clothing without shoes to the nearest 0.1 kg. Body mass index (BMI) was calculated as weight (kilograms)/height (meters) squared.

### Blood sample collection and laboratory methods

After 10–12 h of fasting, a venous blood sample was obtained from each participant. Serum or plasma samples were separated within 15 min of collection, and were analyzed within 30 min or stored at -80 °C for later analysis. Blood samples collected at baseline were used for the measurement of homocysteine (Hcy), folate and lipids levels. Serum lipids including total cholesterol (TC), triacylglycerols (TAG) and high density lipoprotein cholesterol (HDL-C) were performed in the six study center laboratories, using standard reagents and an automatic biochemistry analyzer. Low density lipoprotein cholesterol (LDL-C) was calculated by Friedewald’s equation. Plasma Hcy concentration was determined in duplicate by high-performance liquid chromatography. The intra- and inter-assay coefficients of variation were 3.5 % and 4.2 %, respectively. Serum folate was determined by chemiluminescent immunoassay using a Beckman Coulter ACCESS Immunoassay System (Beckman-Coulter Canada Inc., Mississauga, Canada). The intra- and inter-assay coefficients of variation were 2.3 % and 3.7 %, respectively. All sample collection and tests were performed in an identical manner following the same standard protocol.

### DNA extraction and genotyping

All participants were requested to provide 2 ml peripheral whole blood, which was collected in ethylenediaminetetraacetic acid (EDTA) and stored at -20 °C. Genomic DNA was extracted from the cell pellet in whole blood by the QIAamp Blood Kit (Qiagen, Valencia, California, USA) and evaluate for quality by 1 % agarose gel electrophoresis. TaqMan probe technique was used for detecting gene polymorphisms of the Hcy pathway in the central laboratory. The polymerase chain reaction-restriction fragment length polymorphism (PCR-RFLP) method was applied to detect the *MTHFR* C677T, *MTHFR* A1298C, *MTR* A2756G and *MTRR* A66G genotypes. Universal reaction conditions for each genotyping are as follows: 4 ng dried DNA, 0.08 mL 40 assay locus-specific probe, and 2.0 mL TaqMan universal polymerase chain reaction (PCR) master mix made to a final volume of 4 mL with 1.92 mL of sterile water. Plus the main parameters for the PCR-RFLP of the four single nucleotide polymorphisms (SNPs) are shown in Table 8. The amplified PCR products were separated on 3 % agarose gel. To ensure the accuracy of the genotype, each sample had twice genotyping for the SNP in our present study by two independent researchers. Genotyping call rate for assessments of all genetic variants was ≥ 98 % in this study. After excluding samples that consistently failed, we selected 10 % of total samples for replication, and concordance of 100 % was repeated for all samples.

**Table 8:** Primer sequences and reaction conditions for PCR-RFLP of gene polymorphisms

Gene	Primer sequence	T^a^ and Cycles	Product size	Restriction enzyme
*MTHFR* C677T	Forward: 5’-TGAAGGAGAAGGTGTCTGCGGGA-3’	58 °C, 35	198 bp	*Hinf* I
	Reverse: 5’-AGGACGGTGCGGTGAGAGTG-3’			
*MTHFR* A1298C	Forward: 5’-CTTTGGGGAGCTGAAGGACTACTAC-3’	52 °C, 38	163 bp	*Mbo* II
	Reverse: 5’-CACTTTGTGACCATTCCGGTTTG-3’			
*MTR* A2756G	Forward: 5’-GAACTAGAAGACAGAAATTCTCTA-3’	53 °C, 36	189 bp	*Hae* III
	Reverse: 5’-CATGGAAGAATATCAAGATATTAGA-3’			
*MTRR* A66G	Forward: 5’-GCAAAGGCCATCGCAGAAGACAT-3’	60 °C, 35	151 bp	*Nsp* I
	Reverse: 5’-GTGAAGATCTGCAGAAAATCCATGTA-3’			

^a^Annealing temperature

### Thresholds of dyslipidemia, low folate and high Hcy

According to Chinese Guidelines on Prevention and Treatment of Dyslipidemia in Adults [60], we defined the four types of dyslipidemia as: hypercholesterolemia (serum total cholesterol level > 5.18 mmol/L); hypertriglyceridemia (serum triacylglycerols level > 1.70 mmol/L); Low levels of high-density lipoprotein cholesterol (serum HDL-C level < 1.04 mmol/L); high levels of low-density lipoprotein cholesterol (serum LDL-C level > 3.37 mmol/L). In addition, low folate was considered to be serum folate level lower than 10 nmol/L [61], and high Hcy was defined as homocysteine concentration higher than 15 μmol/L [62, 63].

### Statistical analysis

Statistical analyses were using the IBM SPSS software package (version 19.0 for windows; IBM Inc. Armonk, NY, USA). The results for categorical variables (i.e. sex, clinical centers and genotypes) are presented as number and percentage of cases. Continuous variables (i.e. age, height, weight, body mass index (BMI), systolic blood pressure (SBP), diastolic blood pressure (DBP), folate, Hcy, TC, TAG, HDL-C and LDL-C) were given as the mean ± standard deviation. The means for continuous variables in the two groups were compared using Student’s t tests, and the prevalence of categorical variables was compared using *χ*^2^ tests. Hardy-Weinberg equilibrium for genotypic frequencies of four genes was assessed with the *χ*^2^ test and Fisher’s exact test. Unitary linear regression model was used to access the associations of folate, Hcy and gene polymorphisms on serum lipids levels. Unconditional logistic regression was performed to estimate the independently effects of folate, Hcy status and genotypes on dyslipidemia risk. The interactions of folate, Hcy and genotypes on the incidence of dyslipidemia were also adopted the unconditional logistic regression model and used trend test for further verification. Since the number of cases was small, we adopted the stepwise logistic regression method (Backward: Wald). All analysis were adjusted potential confounding factors, including sex, age, clinical centers, height and weight. A two-sided P value < 0.05 was considered as significant.

## Abbreviations

MTHFR: Methylenetetrahydrofolate reductase
MTR: Methionine synthase
MTRR: Methionine synthase reductase
Hcy: Homocysteine
TC: Total cholesterol
TAG: Triacylglycerols
HDL-C: High-density lipoprotein cholesterol
LDL-C: Low-density lipoprotein cholesterol
BMI: Body mass index
SBP: Systolic blood pressure
DBP: Diastolic blood pressure
